# Contact Reactive Brazing of TC4 Alloy to Al7075 Alloy with Deposited Cu Interlayer

**DOI:** 10.3390/ma14216570

**Published:** 2021-11-01

**Authors:** Mengjuan Yang, Chaonan Niu, Shengpeng Hu, Xiaoguo Song, Yinyin Pei, Jian Zhao, Weimin Long

**Affiliations:** 1 School of Materials Engineering, Shanghai University of Engineering Science, Shanghai 201620, China; yang13137760150@163.com (M.Y.); zhaojianhit@163.com (J.Z.); 2 State Key Laboratory of Advanced Welding and Joining, Harbin Institute of Technology, Harbin 150001, China; ncnhitwh@163.com (C.N.); sp_hu@hit.edu.cn (S.H.); 3 Shandong Institute of Shipbuilding Technology, Weihai 264209, China; 4 State Key Laboratory of Advanced Brazing Filler Metals and Technology, Zhengzhou Research Institute of Mechanical Engineering Co., Ltd., Zhengzhou 450001, China; balloy@163.com (Y.P.); brazelong@163.com (W.L.)

**Keywords:** Al7075, TC4, contact reactive brazing, Cu deposited

## Abstract

The brazing of Titanium alloy to Aluminum alloy is of great significance for lightweight application, but the stable surface oxide film limits it. In our work, the surface oxide film was removed by the ion bombardment, the deposited Cu layer by magnetron sputtering was selected as an interlayer, and then the contact reactive brazing of TC4 alloy to Al7075 alloy was realized. The microstructure and joining properties of TC4/Al7075 joints obtained under different parameters were observed and tested, respectively. The results revealed that the intermetallic compounds in the brazing seam reduced with the increased brazing parameters, while the reaction layer adjacent to TC4 alloy continuously thickened. The shear strength improved first and then decreased with the changing of brazing parameters, and the maximum shear strength of ~201.45 ± 4.40 MPa was obtained at 600 °C for 30 min. The fracture path of TC4/Al7075 joints changed from brittle fracture to transgranular fracture, and the intergranular fracture occurred when the brazing temperature was higher than 600 °C and the holding time exceeded 30 min. Our work provides theoretical and technological analyses for brazing TC4/Al7075 and shows potential applications for large-area brazing of titanium/aluminum.

## 1. Introduction

Ti-6Al-4V (TC4) alloy has unique properties such as low thermal conductivity, superior corrosion resistance, superior mechanical properties, high-temperature strength, and low-temperature toughness, which has attracted wide attention in the aerospace field [1,2,3,4]. Al7075 alloy, which has low density, high specific strength, casting properties, good corrosion resistance, and high conductivity, has been widely used in structural parts of the aerospace field [5,6,7]. Currently, TC4 and Al7075 alloys are simultaneously used in composite components of aircraft wings and automotive airfoils, where the performance of the components can be improved by combining the advantages of the two materials [8,9,10,11,12].

At present, the methods of joining Al alloys to TC4 alloys mainly include laser welding [13,14], transient liquid phase (TLP) bonding [15], brazing [16], diffusion bonding [17,18], etc. Among them, brazing is suitable for joining dissimilar materials with a large difference in physical and chemical properties [9]. For example, Lee et al. [19] brazed Ti alloy and Al alloy using AlSi_10_Mg filler. Chang et al. [20] brazed the Al6061 using Al-10.8Si-10Cu and Al-9.6Si-20Cu at 560 °C, and the results showed that the liquid phase line temperature changed from 592 °C to 570 °C when 10 wt. % Cu was added to the Al-12Si. Contact reactive brazing is a kind of brazing method without any brazing flux [21], which has been widely applied for brazing Al alloys to other alloys, such as Al6063 [22], Al6061 to AZ31B Mg alloy [23], and Al6063 to 1Cr18Ni9Ti stainless steel [24]. Schällibaum et al. [25] studied the microstructure of the AA6082 brazed joints with plating copper, and the results showed that the formation of defects was caused by the residual oxide films aggregated in the brazed joint. Wu et al. [24] used Cu as an interlayer to join Al6063 and 1Cr18Ni9Ti stainless steel by contact reactive brazing. In fact, the existence of an oxide film on the surface of the aluminum alloy and titanium alloy prevented the diffusion and reaction during the brazing process, which deteriorated the interfacial microstructure and then reduced the joining properties [26]. Therefore, the appropriate surface treatment method should be adopted to remove the stable oxide film. As a method of surface modification, ion bombardment can effectively remove the oxide film [27,28], and our previous work also demonstrated it [26]. To prevent re-oxidation after the ion bombardment process, it was chosen that the Cu layers be prepared on their surfaces by magnetron sputtering for protection, as well as that the eutectic reaction between copper and aluminum would occur at 548 °C, which facilitated brazing of the contact reaction between TC4 and Al7075 at a relatively low temperature [29,30].

Based on our previous study, the combination of ion bombardment and magnetron sputtering copper deposition method was used to braze TC4 and Al7075 dissimilar alloys. The microstructural evolution of TC4/Al7075 brazed joints was discussed in detail under different brazing parameters (brazing temperature and holding time), and the TC4/Al7075 brazing processes were optimized based on the joining properties.

## 2. Experimental Procedures

The TC4 and Al7075 alloys were cut in a size of 15 mm × 10 mm × 5 mm and 8 mm × 8 mm × 5 mm, respectively. The brazing surfaces of the TC4 alloy and Al7075 alloy were ground with metallographic sandpaper and polished using a diamond agent down to 2.5 μm. Finally, the polished TC4 alloy and Al7075 alloy were cleaned with acetone under an ultrasonic bath and then air dried. The microstructure of the TC4 and Al7075 alloys are shown in Figure S1.

**Figure 1:**
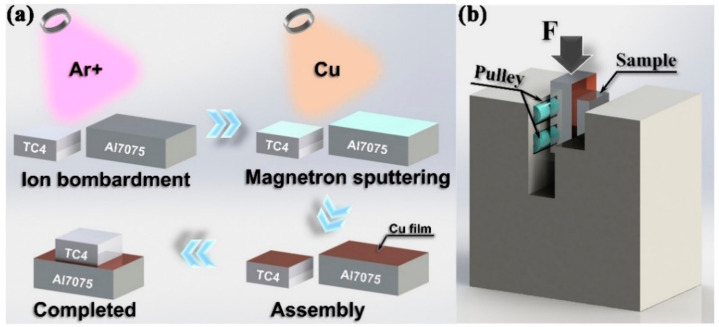
Schematic diagram of the TC4/Al7075 contact reactive brazing process (**a**) and shear test experiment (**b**).

Figure 1a shows the schematic diagram of the entire process. The surface oxide film on the faying surfaces of the TC4 and Al7075 substrates was removed by Ar ion bombardment, and then a Cu layer with a thickness of 5 μm was deposited onto both sides of the brazing surface by magnetron sputtering [31,32]. Subsequently, the brazing process of Al7075 to TC4 was carried out in the furnace of a vacuum level of less than 5.0 × 10^−3^ Pa under the pressure of 0.25 MPa. For the brazing process, all assemblies were heated first to 535 °C at a heating rate of 10 °C/min, kept for 5 min, and then continually heated to the specified brazing temperature (560–620 °C) at a heating rate of 5 min/°C. Subsequently, the brazing samples were held for 15–60 min. Finally, the furnace was slowly cooled to room temperature.

The cross-sectional microstructure of the TC4/Al7075 joints was characterized by the field emission scanning electron microscopy (SEM, MERLIN Compact, Zeiss), energy dispersive spectrometer (EDS, OCTANE PLUS, EDAX), and X-ray diffraction (XRD, JDX-3530M). The shear strength of the TC4/Al7075 joints was tested at a constant rate of 0.5 mm/min by using a universal testing machine (Instron 5967) at room temperature (Figure 1b). The fracture mode and microstructure were analyzed using SEM equipped with EDS, and the phase of the fracture surfaces was identified by XRD.

## 3. Results and Discussion

### 3.1. Typical Microstructure of TC4/Cu Layer/Al7075 Brazed Joint

**Figure 2:**
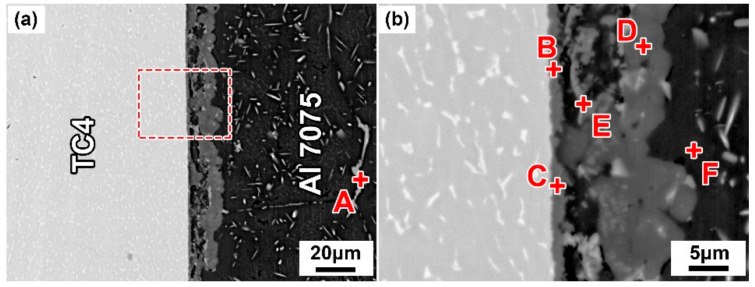
Interfacial microstructure of TC4/Cu layer/Al7075 alloy brazed joint at 600 °C for 30 min. (**a**) Low magnification; (**b**) High magnification

The typical microstructure of the TC4/Cu layer/Al7075 brazed joint at 600 °C for 30 min is shown in Figure 2. It can be seen that the Cu layer reacted completely with the base materials, and a sound joint was formed without any crack or void, as shown in Figure 2a. After the eutectic reaction between the Cu layer and the Al alloy, the resulting eutectic liquid phase penetrated the Al7075 substrate, and many intermetallic compounds (IMCs) were formed in the brazing seam. Spots A, B, C, D, E, and F represent the different phases of the brazed joint in Figure 2, and Table 1 shows the phase compositions of different spots determined by EDS. The atomic ratio of Al and Cu was 2:1 in spots A and E, which may be the Al_2_Cu phase. The atomic ratio of Al and Ti was 3:1 in spot B, revealing the possible formation of the Al_3_Ti phase based on the Al-Ti phase diagram (Figure S2) [33]. The atomic percent proportion of Al and Ti was approximately 5: 3 in spot C, which was confirmed as the Al_5_Ti_3_ phase [34]. Spot D with atomic percent proportion of Al, Cu, and Mg was approximately 2:1:1, which was speculated as the Al_2_CuMg phase according to the Al-Cu-Mg ternary phase diagram (Figure S3) [35]. Spot F was analyzed as the Al-based solid solution (Al(s, s)) according to the EDS result.

**Figure 3:**
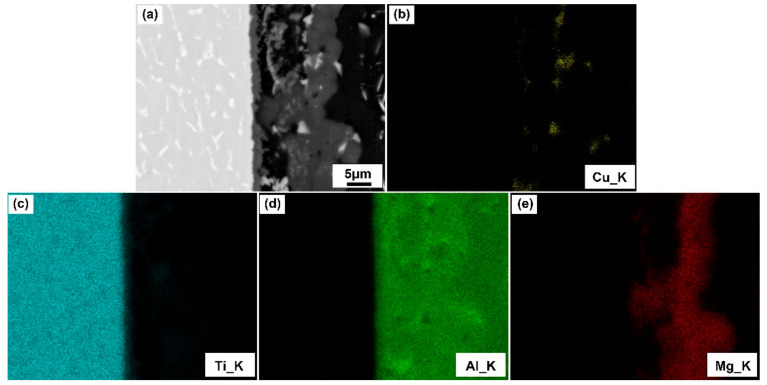
Elemental distribution of TC4/Cu layer/Al7075 brazed joint brazed at 600 °C for 30 min. (**a**) BSE image of the typical brazed joint and the elemental distribution of (**b**) Ti; (**c**) Al; (**d**) Cu; (**e**) Mg.

The elemental distribution of the TC4/Al7075 joint is shown in Figure 3b–e. It can be seen that the substrate gradually dissolved into the eutectic liquid phase as the Al-Cu eutectic phase reacted and spread on the surface of Al alloy substrate during the brazing process. In addition, Figure 3b,e shows the concentrated distribution of Cu and Mg elements in the brazing seam. Okamoto et al. [36] demonstrated that Cu diffused into the Al substrate and formed Al_2_Cu, which was also demonstrated from the Al-Cu binary phase diagram (Figure S4). Meanwhile, Figure 3e shows that the distribution of the Mg element primarily concentrated in the brazing seam and then formed the Al_2_CuMg phase [15,32]. Liu et al. [37] confirmed the interface energy of Al_3_Ti was the lowest, and preferentially formed on the Al substrate. In addition, the metastable intermediate phase of Al_5_Ti_3_ was formed during the reaction [38]. Therefore, the typical interfacial microstructure of the TC4/Al7075 joint brazed at 600 °C for 30 min was TC4 substrate/Al_3_Ti + Al_5_Ti_3_/Al_2_Cu + Al_2_CuMg/Al7075 substrate.

### 3.2. Effect of Brazing Parameters on the Microstructure of TC4/Cu Layer/Al7075 Brazed Joints

**Figure 4:**
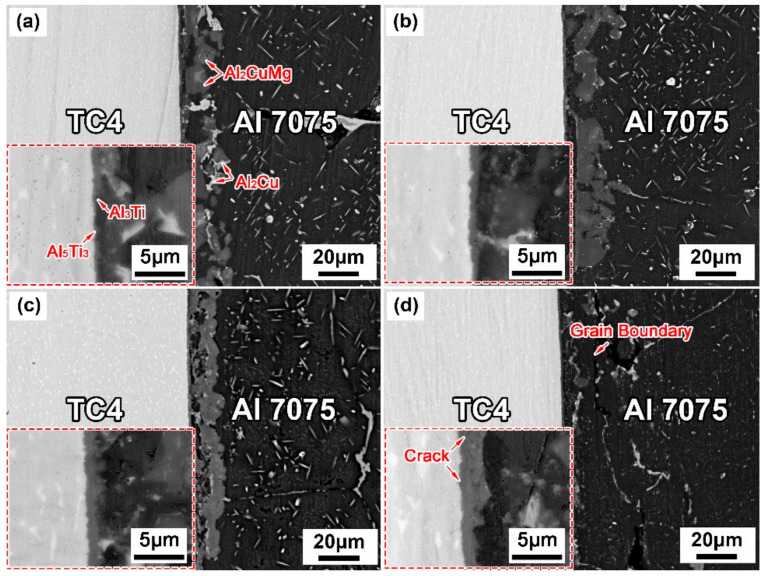
Interfacial microstructures of brazed joints at different temperatures for 30 min. (**a**) 560 °C; (**b**) 580 °C; (**c**) 600 °C; (**d**) 620 °C

**Figure 5:**
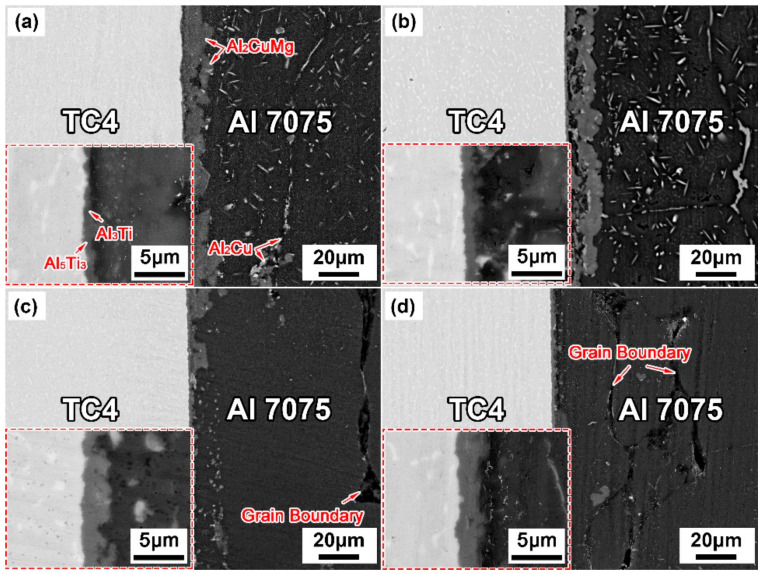
Interfacial microstructures of brazed joints at 600 °C for different holding times. (**a**) 15 min; (**b**) 30 min; (**c**) 45 min; (**d**) 60 min

Figure 4 and Figure 5 show the interfacial microstructures of the TC4/Cu layer/Al7075 brazed joints at various brazing parameters. All brazing temperatures were higher than the Al-Cu eutectic temperature (548 °C). When the brazing temperature was 560 °C (Figure 4a), large amounts of the Al_2_Cu and Al_2_CuMg phases formed in the brazing seam. With an increasing brazing temperature, the formed Al_2_Cu and Al_2_CuMg IMCs gradually decreased and disappeared due to the rapid diffusion of Cu atoms into Al7075 substrate. However, the grain coarsening of the Al7075 substrate appeared when the brazing temperature was 620 °C. When the temperature range was 560–620 °C, it is worth noting that the eutectic liquid phase mainly infiltrated along the Al grain boundaries, and the Al_2_Cu phase formed at the grain boundaries of Al7075.

Figure 5 illustrates the microstructural evolution of the brazed joints with prolonging the holding time. Insufficient diffusion of Cu led to the formation of large and continuous Al_2_Cu and Al_2_CuMg IMCs in the brazing seam at the short holding time (15 min). With the extension of the holding time (30 min), Cu atoms diffused fully into the Al substrates, which caused the decrease of IMCs. With the holding time further raised to 45 min or 60 min, the IMCs substantially reduced in the brazing seam, followed by the grain coarsening of Al7075, which worsened the properties of Al alloy.

**Figure 6:**
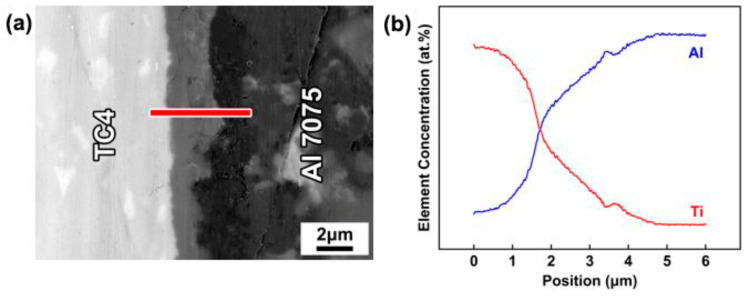
**(a)** Interfacial microstructure of brazed joint, **(b)** the EDS line scanning distribution of Ti and Al elements.

In addition, as shown in Figure 4 and Figure 5, a discontinuous reaction layer of thickness of less than 1 μm was formed on the TC4 side when the brazing parameters were insufficient. As the brazing parameters were raised, the intermetallic compounds’ layer became thicker and more continuous. However, the microcracks appeared in the reaction layer when the brazing parameters were too high (brazing temperature ~ 620 °C and holding time 45–60 min), and it was presumed that it was caused by the difference in thermal expansion coefficient between the reaction layer and the base material. The corresponding EDS line scan results (Figure 6) indicated that the Al and Ti atoms diffused each other to form the diffusion layer.

Based on the above analyses on the interfacial microstructure of the joints with different brazing parameters, the evolution of the TC4/Al7075 brazed joints can be proposed as follows. The Al-Cu eutectic liquid phase was formed when the brazing temperature exceeded the Al-Cu eutectic temperature of 548 °C. As the brazing parameters increased, the Cu atoms fully diffused into the substrate and reacted with Al to produce more eutectic liquid phase. Meanwhile, the Mg atoms from the substrate entered the liquid and reacted with Al and Cu atoms to form Al_2_CuMg. The residual liquid solidified and formed the eutectic structure (α-Al + Al_2_Cu) in the brazing seam during the cooling stage [32]. On the other hand, the formation of the Al-Cu eutectic liquid phase could promote the diffusion of Ti atoms into the liquid phase and produce the diffusion gradient on the TC4 side. The Al and Ti elements reacted and formed Al_3_Ti according to the Al-Ti binary phase diagram [15]. Moreover, the metastable intermediate phase of Al_5_Ti_3_ was formed in the interface during the reaction, owing to insufficient atomic diffusion [38].

### 3.3. Effect of Brazing Parameters on the Mechanical Properties of Brazed Joints

**Figure 7:**
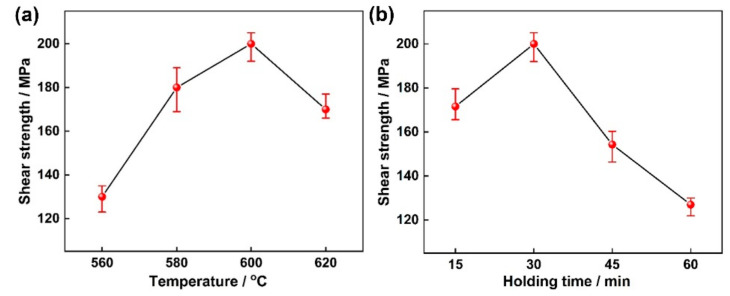
Effect of the brazing parameters on mechanical properties of brazed joints. (**a**) Brazing temperature; (**b**) Holding time

Figure 7 shows the shear strength of the TC4/Cu layer/Al7075 brazed joints at various brazing parameters. The shear strength improved first and then decreased evidently, and the maximum shear strength of ~201.45 ± 4.4 MPa was obtained at 600 °C for 30 min. Combined with the analysis of the interfacial microstructures, large amounts of brittle intermetallic compounds of the Al_2_Cu and Al_2_CuMg phase distributed continuously in the brazing seam when the brazing temperature was low (560 °C), which deteriorated the joining properties. The enhanced diffusion ability of the Cu layer led to the reduction of Al-Cu IMCs, and the composition of the brazing seam tended to be uniform at a brazing temperature of 600 °C and a holding time of 30 min, which enhanced the joint strength effectively. However, with a further increase of the brazing temperature (620 °C) and holding time (45 min and 60 min), the sufficient diffusion of Cu atoms resulted in the decrease of the Al-Cu intermetallic compound, and the grain growth of the Al substrate and the formed microcracks at grain boundaries had a detrimental effect on the shear strength.

**Figure 8:**
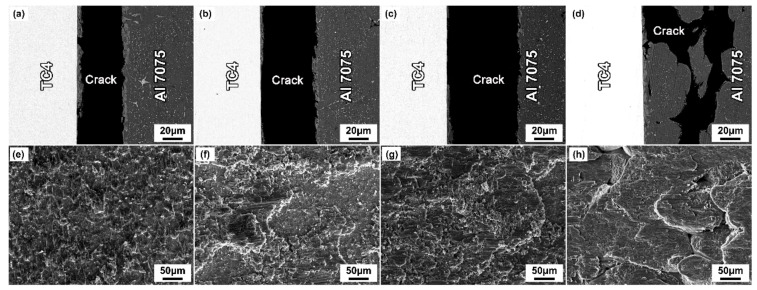
Fracture morphologies of TC4/Cu layer/Al7075 joints brazed at different temperatures for 30 min. (**a,e**) 560 °C; (**b**,**f**) 580 °C; (**c**,**g**) 600 °C; (**d**,**h**) 620 °C.

**Figure 9:**
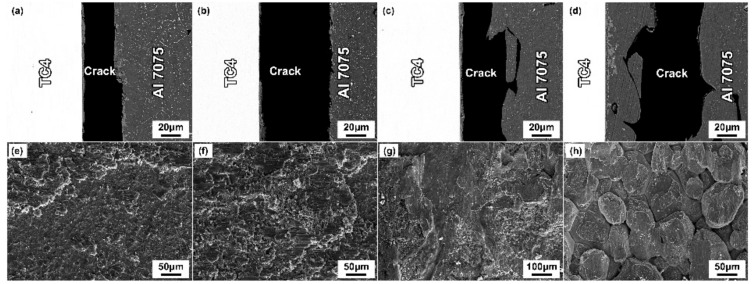
Fracture morphologies of TC4/Cu layer/Al7075 joints brazed at 600 °C for different times. (**a**,**e**) 15 min; (**b**,**f**) 30 min; (**c**,**g**) 45 min; (**d**,**h**) 60 min.

**Figure 10:**
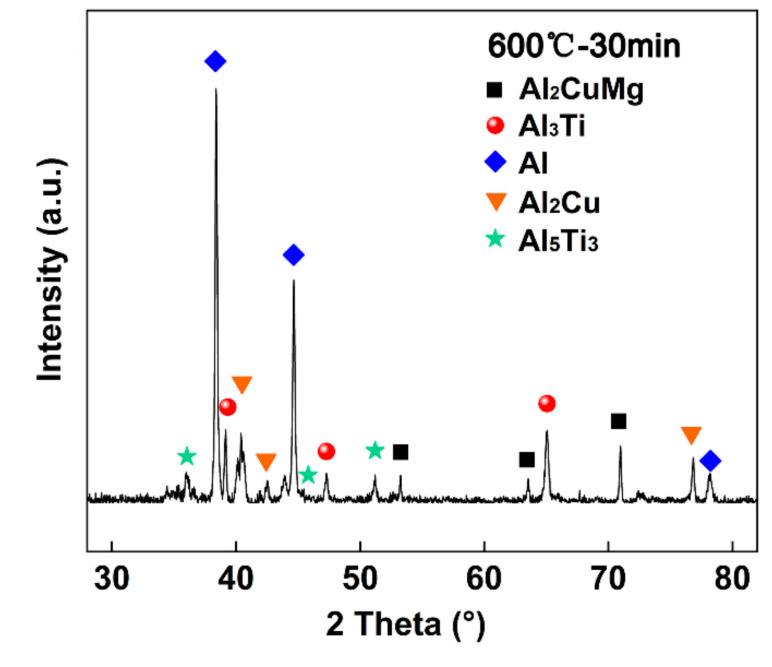
XRD pattern of the fracture surface (Al7075 side).

To further analyze the effect of the brazing parameters on the fracture path and fracture mode of the joints, the fracture analysis of the joints was performed after the properties’ test, as shown in Figure 8 and Figure 9. As the brazing parameters increased, the cracks extended mainly along the intermetallic compounds. The XRD pattern of the fracture surface (Figure 10) showed the presence of Al_2_Cu, Al_2_CuMg, Al_5_Ti_3,_ and Al_3_Ti phases, which was consistent with the above results. With a further increase of the brazing parameters, the Al-Cu intermetallic compounds decreased gradually, and the fracture path mainly propagated along the intermetallic compounds in the brazing seam. Except for that, part of the cracks propagated inside the Al grains and a transgranular fracture formed. With the continuous elevation of the brazing parameters, the contents of Al_2_Cu and Al_2_CuMg intermetallic compounds decreased gradually. Meanwhile, the cracks propagated inside the Al substrates and IMCs. When the brazing parameters were too high (above 600 °C), the fracture extended along the Al grain and intergranular fracture happened.

## 4. Conclusions

1. The contact reactive brazing of the TC4 alloy to the Al7075 alloy was achieved using deposited Cu as an interlayer. The typical interfacial microstructure of the TC4/Al7075 brazed joint was the TC4 substrate/Al_3_Ti + Al_5_Ti_3_/Al_2_Cu + Al_2_CuMg/Al7075 substrate at 600 °C for 30 min.
2. With increasing the brazing temperature and holding time, the amount of Al_2_Cu and Al_2_CuMg IMCs in the brazed joints decreased and the homogenization of the joint composition improved, while the thickness of the reaction layer (Al_3_Ti + Al_5_Ti_3_) on the TC4 side increased gradually.
3. The shear strength improved first and then decreased with increasing brazing parameters, and the maximum shear strength of ~201.45 ± 4.40 MPa was obtained at 600 °C for 30 min. The fracture mode of the joint changed from brittle fracture to transgranular fracture, and the intergranular fracture occurred when the brazing temperature was higher than 600 °C and the holding time exceeded 30 min.

## Figures and Tables

**Table 1:** EDS analysis of the selected spots in Figure 2 (at. %).

Spot	Al	Ti	Cu	Mg	Possible Phase
A	65.64	0.92	25.08	8.36	Al_2_Cu
B	71.95	26.45	0.54	1.06	Al_3_Ti
C	60.51	36.98	1.20	1.31	Al_5_Ti_3_
D	61.84	3.80	16.24	18.12	Al_2_CuMg
E	63.60	2.30	28.57	5.53	Al_2_Cu
F	96.47	1.22	1.17	1.14	Al(s, s)

## Data Availability

The raw/processed data in the paper cannot be shared at present owing to part of an ongoing further study.

## Supplementary Materials

The following are available online at https://www.mdpi.com/article/10.3390/ma14216570/s1, Figure S1: the characterization microstructures of the TC4 and Al7075 substrate: (a) TC4; (b) Al7075, Figure S2: Al-Ti binary phase diagram, Figure S3: Al-Cu-Mg ternary phase diagram, Figure S4: Al-Cu binary phase diagram.
