# Supplementary material for: Contact Reactive Brazing of TC4 Alloy to Al7075 Alloy with Deposited Cu Interlayer
Source: Materials (Basel). 2021 Nov 1;14(21):6570. doi: 10.3390/ma14216570 (PMC8585328; doi:10.3390/ma14216570)
Supplement: Supplementary file 1 [file materials-14-06570-s001.zip › materials-1405043-supplementary.pdf]

materials

Contact Reactive Brazing of TC4 Alloy to Al7075 Alloy with Deposited Cu Interlayer

Mengjuan Yang [1], Chaonan Niu [2], Shengpeng Hu [2], Xiaoguo Song [2,3,*], Yinyin Pei [4], Jian Zhao [1] and Weimin Long [4]

[1] School of Materials Engineering, Shanghai University of Engineering Science, Shanghai 201620, China; yang13137760150@163.com (M.Y.); zhaojianhit@163.com (J.Z.)
[2] State Key Laboratory of Advanced Welding and Joining, Harbin Institute of Technology, Harbin 150001, China; ncnhitwh@163.com (C.N.); sp_hu@hit.edu.cn (S.H.)
[3] Shandong Institute of Shipbuilding Technology, Weihai 264209, China
[4] State Key Laboratory of Advanced Brazing Filler Metals and Technology, Zhengzhou Research Institute of Mechanical Engineering Co., LTD, Zhengzhou 450001, China; balloy@163.com (Y.P.); brazelong@163.com (W.L.)
* Correspondence: xgsong@hitwh.edu.cn; Tel./Fax: +86-631-5678454

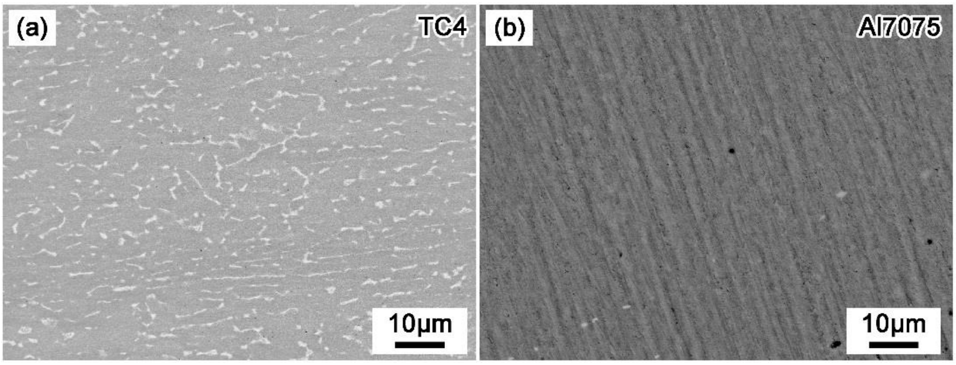

Figure S1. the characterization microstructures of the TC4 and Al7075 substrate: (a) TC4; (b) Al7075.

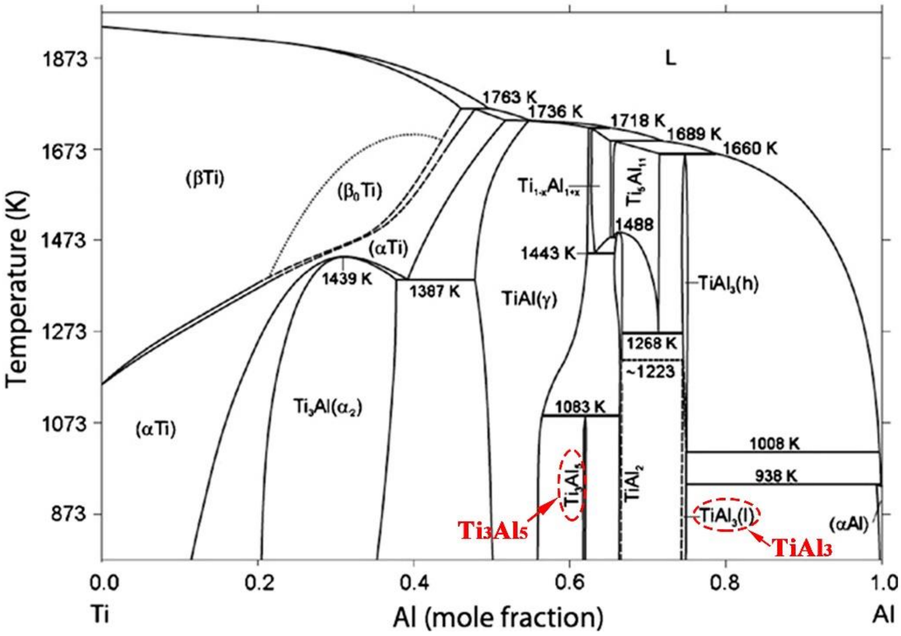

Figure S2. Al-Ti binary phase diagram.

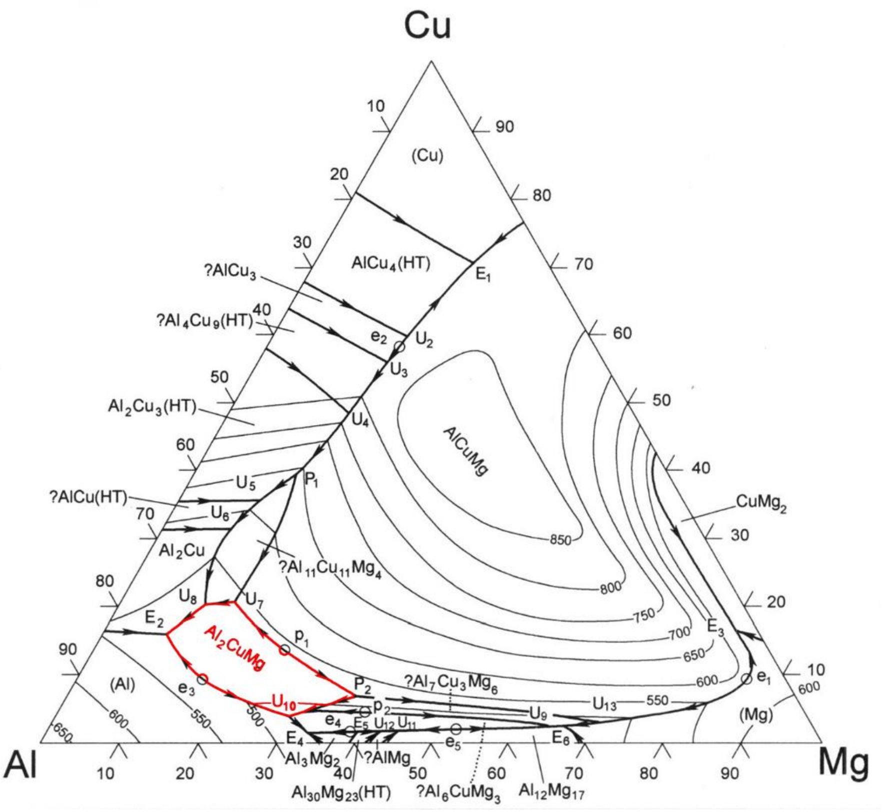

Figure S3. Al-Cu-Mg ternary phase diagram.

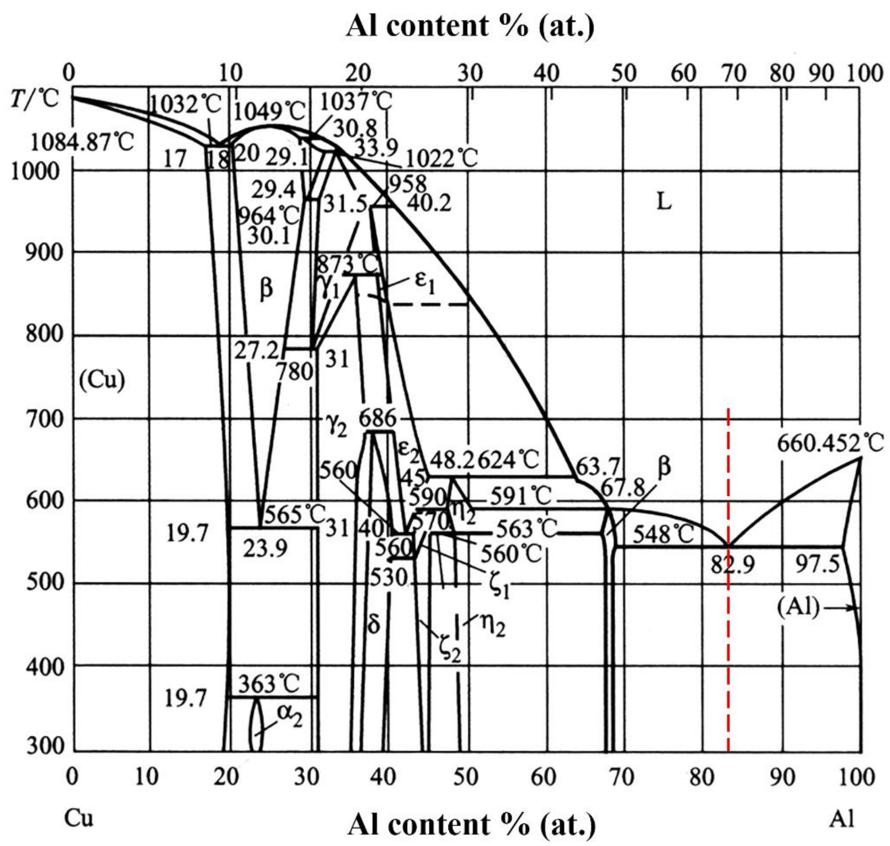

Figure S4. Al-Cu binary phase diagram.
